# Plant-Adapted *Escherichia coli* Show Increased Lettuce Colonizing Ability, Resistance to Oxidative Stress and Chemotactic Response

**DOI:** 10.1371/journal.pone.0110416

**Published:** 2014-10-14

**Authors:** Maria de los Angeles Dublan, Juan Cesar Federico Ortiz-Marquez, Lina Lett, Leonardo Curatti

**Affiliations:** 1 Instituto de Investigaciones en Biodiversidad y Biotecnología, Consejo Nacional de Investigaciones Científicas y Técnicas, Mar del Plata, Buenos Aires, Argentina; 2 Fundación para Investigaciones Biológicas Aplicadas, Mar del Plata, Buenos Aires, Argentina; 3 Laboratorio Integrado de Microbiología Agrícola y de Alimentos, Facultad de Agronomía, Universidad Nacional del Centro de la Provincia de Buenos Aires, Azul, Buenos Aires, Argentina; University of the West of England, United Kingdom

## Abstract

**Background:**

*Escherichia coli* is a widespread gut commensal and often a versatile pathogen of public health concern. *E. coli* are also frequently found in different environments and/or alternative secondary hosts, such as plant tissues. The lifestyle of *E. coli* in plants is poorly understood and has potential implications for food safety.

**Methods/Principal Findings:**

This work shows that a human commensal strain of *E. coli* K12 readily colonizes lettuce seedlings and produces large microcolony-like cell aggregates in leaves, especially in young leaves, in proximity to the vascular tissue. Our observations strongly suggest that those cell aggregates arise from multiplication of single bacterial cells that reach those spots. We showed that *E. coli* isolated from colonized leaves progressively colonize lettuce seedlings to higher titers, suggesting a fast adaptation process. *E. coli* cells isolated from leaves presented a dramatic rise in tolerance to oxidative stress and became more chemotactic responsive towards lettuce leaf extracts. Mutant strains impaired in their chemotactic response were less efficient lettuce colonizers than the chemotactic isogenic strain. However, acclimation to oxidative stress and/or minimal medium alone failed to prime *E. coli* cells for enhanced lettuce colonization efficiency.

**Conclusion/Significance:**

These findings help to understand the physiological adaptation during the alternative lifestyle of *E. coli* in/on plant tissues.

## Introduction

*Escherichia coli* is a common resident of animal hosts mostly as a commensal of the lower intestine of mammals. However, some strains are versatile pathogens of vertebrates, thought to kill more than 2 million humans per year through both intraintestinal and extraintestinal diseases, some of them at infective doses as low as ten cells [1]. Among them, *E. coli* O157:H7 has been a serious public-health concern worldwide since the first outbreak report in 1982 [2].

It is presumed that about one-half of the total population of *E. coli* resides in the primary habitat of the host (lower intestine of warm blood animals) and the other half is in the external environment (secondary habitat) [3]. It has been traditionally accepted that *E. coli* grows and divides in its primary habitat but does not live in non-host environments. Thus, continuous bulk transfer from humans and animals would account for a stable population in the secondary hosts or habitats. This thought became the basis for the use of coliforms determination as an indication of fecal contamination [3].

However, several *E. coli* strains are members of the natural flora in certain tropical ecosystems in the absence of known fecal contamination [3]–[5]. *Escherichia coli* internalization, and often colonization, of vegetables such as lettuce [6], spinach [7], apple [8], orange [9], cress and radish [7], carrots and onions [10] constitutes a serious risk for the health of fresh produce consumers since most commonly used sanitizers are less effective under these circumstances [11]. The factors that allow *E. coli* strains to persist in a non-host environment such as plant tissues remain poorly understood [12], [13]. The available datasets of genomic information from plant associated isolates and its comparison to their corresponding close relatives displaying an animal host preference has shed some valuable insights into the endophytic lifestyle of *E. coli*. Thus, it has been proposed that similar strategies comprising initial adherence, invasion, and establishment are required for bacteria to colonize any host, whether plant or animal [14].

In this work we characterized physiological adaptations during the alternative lifestyle of *E. coli* in/on plant tissues. We show that bacteria isolated from leaves are more efficient plant colonizers, more tolerant to oxidative stress and present a more stringent chemotactic response to leaf extracts. These findings support and improve the current understanding of *E. coli* lifestyle in/on plants.

## Results

### *Escherichia coli* Colonization of Lettuce

A gnotobiotic experimental system comprising lettuce seedlings transplanted onto agarized-Hoagland medium (0.6% agar-agar) inoculated with *E. coli* K12 strain MG1655 was used to study basic aspects of *E. coli* internalization and multiplication in/on plants as a non-host environment. Using the standardized colonization assay, *E. coli* colonized lettuce roots and leaves at an average of 6.9×10^9^ or 7.3×10^7^ CFUs per gram of tissue, respectively. The colonized roots or leaves were rinsed with sterile physiological solution until no CFUs were obtained from the washing solution. This treatment reduced the number of *E. coli* CFUs retrieved from homogenized plant samples by approximately one log.

Inspection of surface-sterilized lettuce seedlings colonized with fluorescent bacteria through the laser confocal microscope confirmed the internalization of *E. coli* cells into the lettuce leaves (Fig. S1 and video S1).

**Figure 1:**
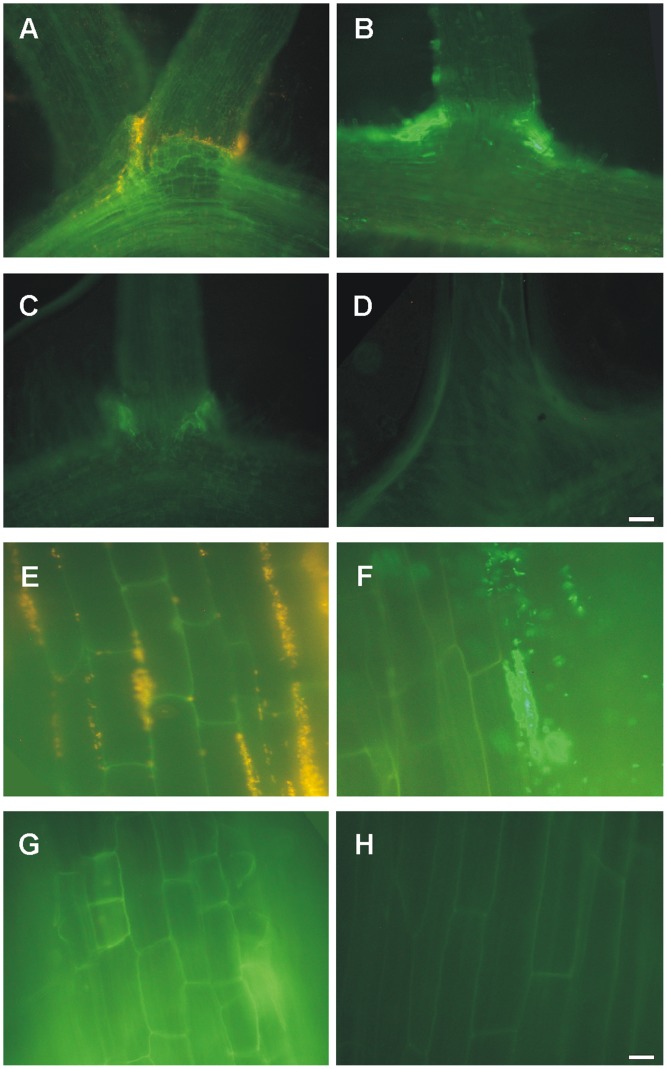
Representative fluorescence photomicrographs of *E. coli* K12-colonized lettuce roots. (A–D) Detail of *E. coli* localization to zones of lateral root emergence or (E–H) root elongation zone after inoculating seedlings with (A and E) RFP-, (B and F) GFP-, (C and G) non-labeled cells or (D and H) non-inoculated seedlings. Magnifications were at (A–D) 150X or (E–H) 750X. The white bar corresponds to 20 µm (A–D) or 10 µm (E–H).

We observed *E. coli* cells in contact with lettuce roots mostly localized to the root hairs outside the plant organ (not shown), and adjacent to some root domes in zones of lateral root emergence (Fig. 1A–D). Inside the root tissue, bacterial cells tended to form clusters in rows, apparently in the apoplastic space between root cells (Fig. 1E–H).

**Figure 2:**
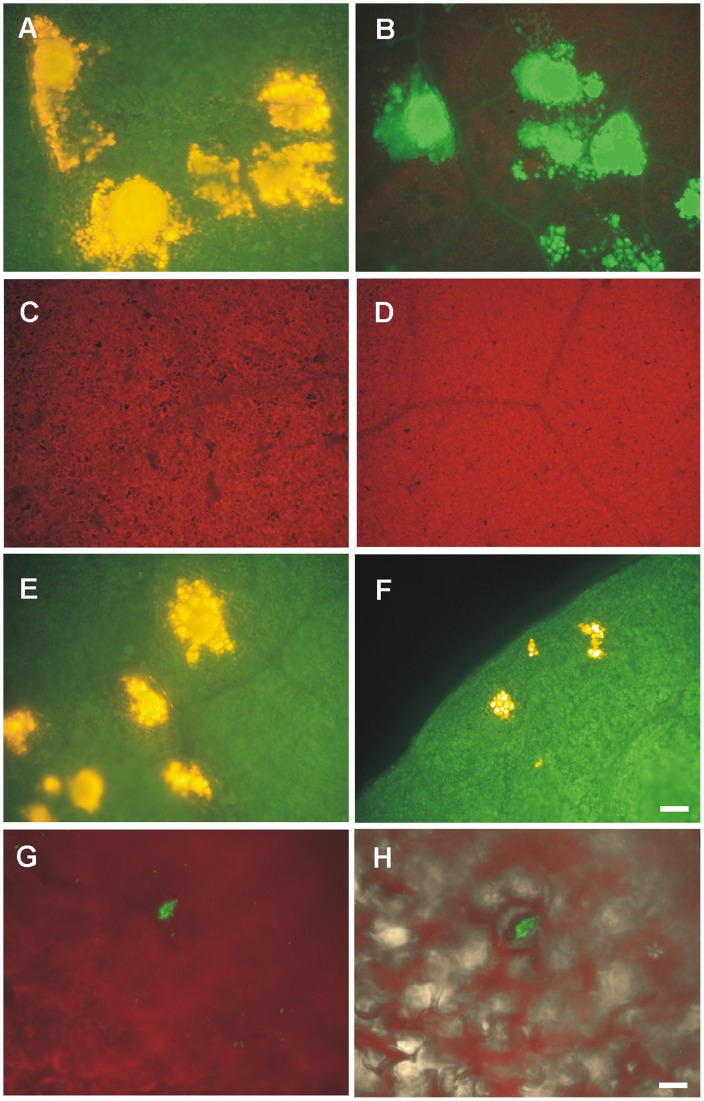
Representative fluorescence photomicrographs of *E. coli* K12-colonized lettuce leaves. Detail of *E. coli* cell aggregates in leaves after the inoculation of seedlings with (A) RFP-, (B) GFP-, (C) non-labeled cells or (D) non-inoculated seedlings. Detail of characteristic bacterial aggregates at (E) leaf base (close to the petiole) or (F) leaf tip (opposite to the petiole margin) of seedlings colonized with RFP-labeled *E. coli*. (G–H) Detail of a leaf from a lettuce seedling that had been superficially inoculated with GFP-labeled *E. coli*. (G) Representative fluorescence photomicrography and (H) merged images of fluorescence and bright field microscopy. Magnifications were at 150X (A–F) or 750X (G–H). White bars correspond to 20 µm (A–F) or 10 µm (G–H).

In/on leaves, patches or aggregates of bacterial cells were observed in close proximity to the vascular tissue. These bacterial cell patches were characteristically larger at the base of leaves than in tips (Fig. 2A–F, Fig. S2A–B). This characteristic pattern of cell patches in close proximity to the vascular tissue was different from that observed when *E. coli* cells were directly spotted onto the leaves surfaces. In this case the bacterial cells remained on the surface of the leaves for a few weeks, mostly in association with the stomata (Fig. 2G–H). Furthermore, *E. coli* cells were efficiently removed by washing when using this inoculation mode (not shown).

**Figure 3:**
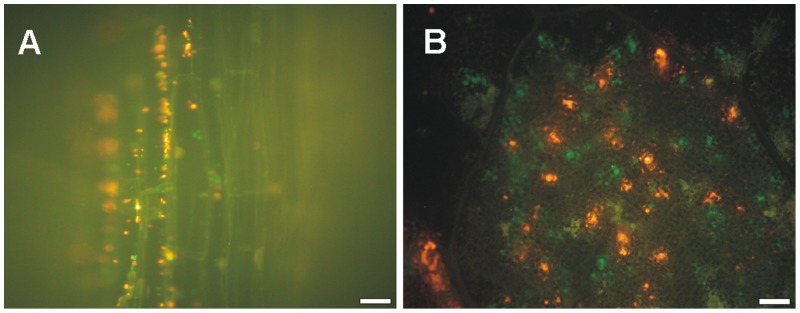
Origin of *E. coli* cell-aggregates in roots or leaves of colonized lettuce seedlings. Representative fluorescence photomicrographs of *E. coli* K12-colonized (A) roots or (B) leaves after the inoculation of seedlings with a 1∶1 mixture of RFP- and GFP-labeled bacteria. Magnifications were at (A) 750X or (B) 150X. White bars correspond to (A) 10 µm or (B) and 20 µm.

To investigate the origin of the *E. coli* cell aggregates we inoculated the Hoagland medium containing 0.6% agar-agar with a 1∶1 mixed population of GFP- and RFP-labeled bacteria. The *E. coli* cell aggregates in/on roots were looser than those of leaves and contained mostly random combinations of both GFP- and RFP-labeled bacteria (Fig. 3A). Conversely, cell aggregates in/on leaves were green or red at a roughly 1∶1 ratio (Fig. 3B).

### *Escherichia coli* Isolated from Lettuce Leaves Are More Efficient Seedlings Colonizers

**Figure 4:**
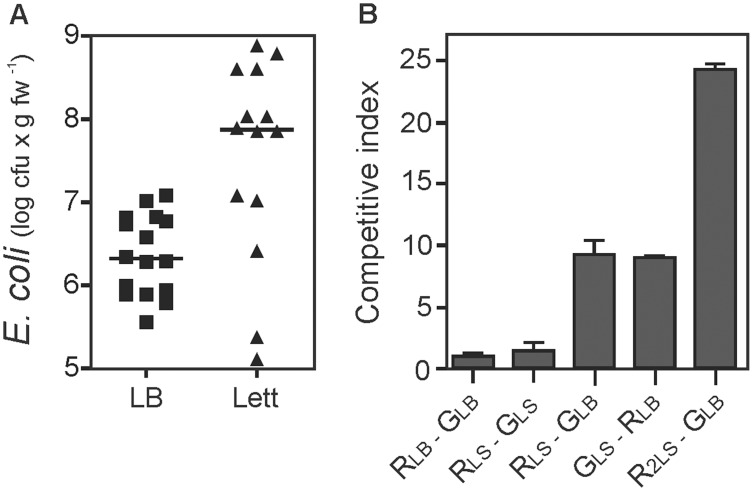
*Escherichia coli* adaptation to lettuce seedlings. (A) Data from individual assays of the colonization of lettuce leaves with non-adapted *E. coli* K12 strain MG1655 cultivated in LB medium (LB) or plant-adapted *E. coli* strain MG1655 freshly isolated from colonized lettuce leaves (Lett). The bars represent the median value of the data sets. (B) Competitive index (CI) analysis of the adaptation of *E. coli* K12 to colonize lettuce seedling. R_LB_ and G_LB_ are non-adapted (cultivated in LB medium) RFP- or GFP-labeled bacteria, respectively. R_LS_ and G_LS_ are RFP- or GFP-labeled, plant-adapted bacteria (isolated from colonized lettuce leaves). R_2LS_ are RFP-labeled bacteria adapted by two consecutive cycles of colonization of lettuce seedlings (isolated from leaves colonized with R_LS_ bacteria). Each data point in (A) (16 for non-adapted bacteria and 14 for adapted bacteria) corresponded to independent assays conducted at 4 to 6 different times over the course of two years. In (B) three assays per condition were conducted at two different times. Each assay (data point) consisted of three seedlings that were inoculated with bacteria in the same test tube. Differences were statistically significant (P≤0.05).

When we used *E. coli-*colonized leaf macerates as a source of bacteria for subsequent rounds of seedlings colonization we observed, on average, an apparent 10-fold increase in the total number of bacteria recovered from the next round of leaves colonization (Fig. 4A). Although the level of colonization appeared to be naturally variable, the observed maximal bacterial counts from lettuce leaves were up to 100-fold higher when inoculated with bacteria isolated from leaves. To further confirm this trait, competence experiments were performed by co-inoculating an equal number of RFP-labeled *E. coli* cells cultured in LB medium (non-adapted cells) and GFP-labeled cells (plant-adapted cells) freshly recovered from a previous colonization assay. Either in this or the reciprocal experiment (GFP-bacterial cells from LB medium and RFP-bacterial cells from leaves) a 9-fold increase in the competitive index towards the plant-acclimated cells was observed. These results indicated no bias according to possible differences in fitness cost due to labelling the bacteria with either fluorescent protein. Moreover, when *E. coli* populations were recovered from leaves after two consecutive cycles of seedlings colonization and then challenged against non-adapted bacteria, an even larger competitive index of 24-fold was observed for this *E. coli* population. Control experiments showed a competitive index for RFP-labeled to GFP-labeled cells of 1.0 (non-adapted) or 1.4 (plant-adapted), respectively. These results fit reasonably well with the expected value (1.0) for both non-competitive bacterial populations (Fig. 4B). When bacteria isolated from leaves were cultivated in LB medium overnight and then used for colonization assays they only partially conserved the enhanced colonization efficiency with a competitive index of 4.0 in comparison with non-adapted bacteria.

Failure to observe any considerable difference between cells cultivated in LB or M9 for non-adapted bacteria suggested that the increase in colonization ability may not be related to the nature or composition of the medium itself (not shown).

### *Escherichia coli* Isolated from Lettuce Leaves Is More Tolerance to Oxidative Stress

**Figure 5:**
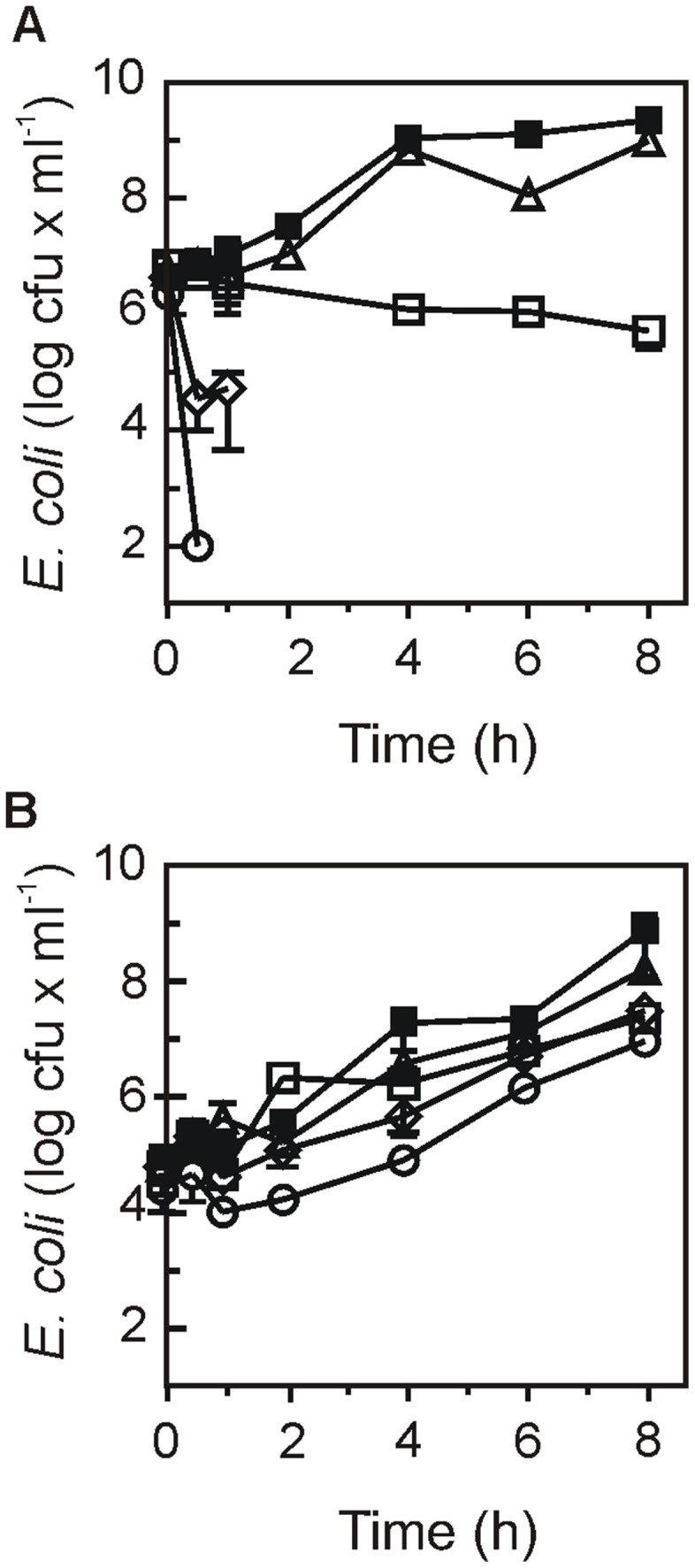
Effect of adaptation to lettuce leaves on *Escherichia coli* K12 tolerance to oxidative stress. Survival of (A) non-adapted bacteria (cultivated in LB medium) or (B) plant-adapted bacteria (isolated from leaves) after incubation for 0 to 8 h in the presence of (▪) 0; (Δ) 0.5; (□) 2; (◊) 8; or (○) 32 mM H_2_O_2_. Data represent the mean and SD of 2 independent assays.

We challenged plant-adapted *E. coli* cells with hydrogen peroxide and observed a dramatic increase in tolerance in comparison with non-adapted bacteria cultivated in M9 medium. Remarkably, cells isolated from leaves still grew at hydrogen peroxide concentrations that largely compromised survival of M9 cultivated cells (Fig. 5A–B).

Since the increased colonization ability correlated with increased oxidative stress tolerance, LB cultivated cells were acclimated to oxidative stress (Fig. S3 A), and then used for lettuce colonization assays. However, these treatments failed to trigger an increased colonizing ability (Fig. S3 B).

### Chemotactic Response of *Escherichia coli* Isolated from Lettuce Leaves

**Figure 6:**
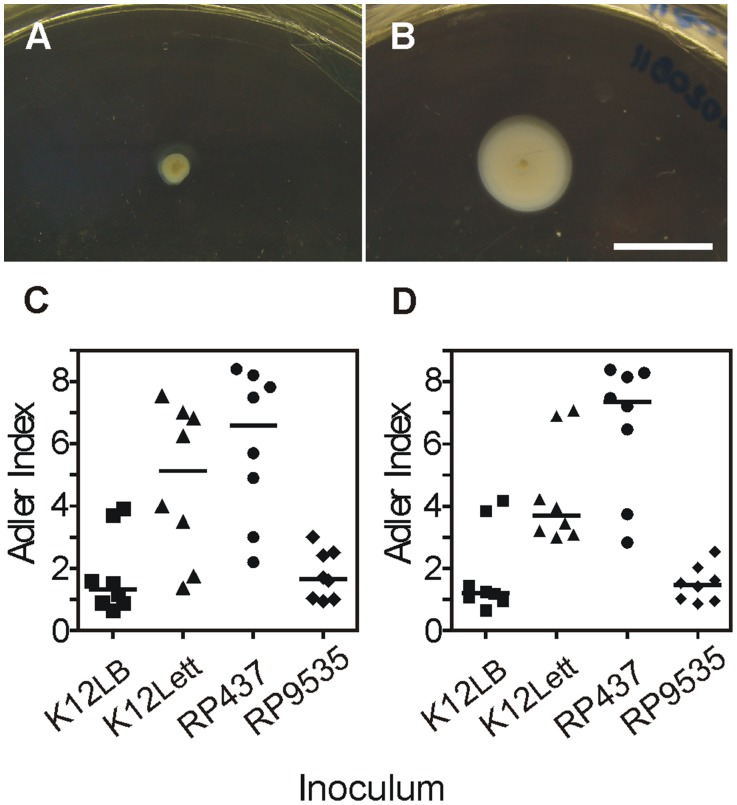
Chemotactic response of plant-adapted *Escherichia coli*. (A–B) Chemotactic response onto TB medium of (A) non-adapted or (B) plant-adapted bacteria. The bar in (A–B) represents 1 cm. (C–D) Chemotactic response towards (C) lettuce leaf blade or (D) lettuce-leaf main vein extracts. K12LB, non-adapted *E. coli* strain MG1655; K12, plant-adapted *E. coli* strain MG1655; RP437, non-adapted *E. coli* strain RP437 (genomic background of reference for chemotactic analysis); and RP9535, non-adapted *E. coli* strain RP9535 (Δ*cheA1643* mutation in a RP437 genomic background). Strains RP437 and RP9535 were used as control of chemotactic and non-chemotactic *E. coli* K12 bacteria. Results in C–D are statistically different (P≤0.05).

Plant-adapted *E. coli* cells presented a considerably more active chemotactic response as observed by migration onto TB plates (Fig. 6A–B). To confirm this result migration towards glass capillaries filled with leaf blade or vascular tissue cell-free extracts was scored as a ratio of migration towards sterile PBS buffer (control) from the same bacterial reservoir. Migration towards lettuce extracts was on average 4- to 5-fold more prominent for plant-adapted than non-adapted bacteria cultivated in LB medium. As a control we show that, conversely to its isogenic line (RP437), a mutant strain impaired in the chemotactic response (*cheA*- Δ*cheA1643* strain RP9535) showed no preferential migration towards leaf-extracts (Fig. 6C–D).

**Figure 7:**
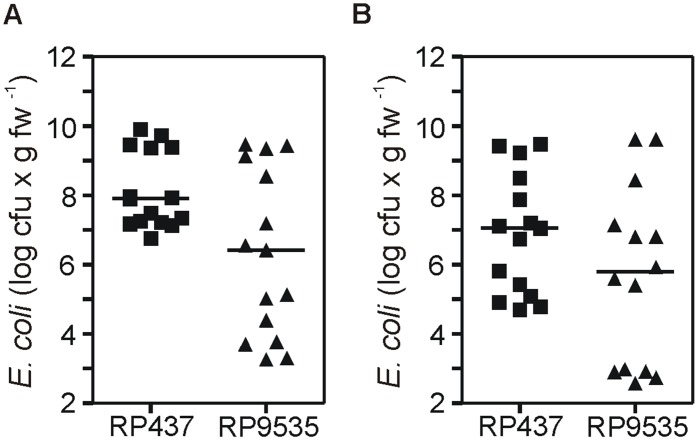
Effect of disrupting the chemotactic response of *Escherichia coli* on the bacterial colonization of lettuce seedlings. (A–B) *E. coli* colonization of lettuce seedlings (A) roots or (B) leaves after inoculation with non-adapted strains RP437 or PR9535. The bars represent the median value of the datasets.

Using the standard colonization assay (10^8^ non-chemotactic cells×mL^−1^ for 20 days), bacterial counts from roots (Fig. 7A) or leaves (Fig. 7B) were on average 15-fold lower in comparison with those reached by a similar inoculum of the chemotactic isogenic strain. Results were highly variable, similarly to those obtained for *E. coli* strain MG1655 colonized seedlings (Fig. 4A). It is noteworthy that the minimal colonization counts observed for the chemotactic cells were up to 1,000-fold higher than those of the non-chemotactic mutant cells (Fig. 7A–B).

In the standard assay used in this study both the inoculum size (Fig. S4 A) and incubation time (Fig. S4 B) were set up in excess to maximize the bacterial colonization of leaves. Thus, to further challenge strains impaired in their chemotactic response, we compared their performance to that of the wild type cells. We applied conditions where excess inoculum size and progression of colonization could not have compensated for some cell defect in colonization ability. Thus, *E. coli* cells were inoculated at 10^5^ cells×mL^−1^ in pools of three seedlings each. After 14 days, all of them showed root colonization and one showed leaf colonization when inoculated with chemotactic bacteria. Conversely, no root or leaf colonization was observed when seedlings were inoculated with non-chemotactic bacteria in a same number of assays, confirming the results obtained by the standard assay. Inoculation with 10^2^ cells of either strain×mL^−1^ failed to produce colonized seedlings in 14 days.

## Discussion

This work describes basic aspects of lettuce colonization by a human commensal strain of *E. coli* K12. The colonization assays that were set up for this research produced lettuce colonization patterns that are in agreement with previous findings. Briefly, *E. coli* (among some other enterobacteria) (*i*) preferentially invade plant root tissue rather than foliage, (*ii*) about 10% of the bacteria are presumably internalized and/or tightly attached to the plant tissues, (*iii*) bacterial invasion of roots is more prominent at lateral root junctions, and in the root cortex apoplast [13]. Using an *in vitro* system similar to the one describe here, Wright et al. [15] showed that *E. coli* MG1655 was able to colonize internally only one lettuce plant out of 10. In contrast 0.5% of *E. coli* (sakai) population was internalized [15]. In field grown leafy greens, including lettuce, *E. coli* internalization and colonization was also shown to be rare [16].

Additionally, our work shows that *E. coli* K12 cells produce large microcolony-like cell aggregates in lettuce leaves that appear to arise from multiplication of single bacterial cells that reach those spots. Such a defined pattern was not observed in roots, suggesting variations of the endophytic lifestyle of *E. coli* when internalized into different plant tissues. It was apparent that while bacteria build up their number by massive multiplication from a rather reduced number of foci (probably single cells) in leaves of colonized seedlings, direct access from the substrate into the roots might account for the observed number and distribution of bacteria in this plant organ.

The characteristic association of bacterial aggregates to the leaves vascular tissue suggests that this might be the point of access of the bacteria from the roots into the leaf tissue. Bulk transfer of bacteria along the surface of the seedlings [17] could be ruled out since surface-inoculation produced a noticeably different colonization pattern. Only very occasionally during this study fluorescent cells were found inside the vascular tissue, suggesting that in the experimental system used *E. coli* propagation and residency might not be prominent in this plant tissue. These results are in agreement with previous studies that show that vascular tissue and xylem cells may be invaded by comparatively lower densities of endophytic bacteria, representing a transit path for systemic spreading into leaves [12]. On the other hand, it has been shown that *Pseudomonas syringae* develops cell aggregates within bean leaves preferentially associated with veins, which has been interpreted as bacteria localizing next to a rich source of nutrients [18]. Thus, these two alternative hypotheses for the localization of cell aggregates close to vascular tissue are not mutually exclusive.

Remarkably, we showed and confirmed by means of reciprocal competitive assays that *E. coli* isolated from colonized leaves progressively colonize lettuce seedlings to higher titers. This result suggests a fast adaptation process probably mediated by the selection of more efficient genotypic/phenotypic variant(s) of the bacterium towards lettuce colonization. Similar results were obtained after sequentially passaging the type strain of *S. enterica* sv. *typhimurium* ATCC14028 through tomatoes. It was shown that spontaneous mutants that arose during tomato passages were 5- to 50-fold more competitive than the wild-type inside tomato fruits but presented a reduced fitness in laboratory medium [19]. In agreement, cultivation in laboratory medium between passages through lettuce leaves decreased the colonization competence of plant-adapted *E. coli* populations, suggesting that these populations might comprise bacteria with contrasting fitness in lettuce seedlings and laboratory medium.

We reasoned that a physiological comparison of plant-adapted vs. non-adapted bacteria would cast some insights into the physiology of the lifestyle of *E. coli* in/on lettuce as an alternative host. We observed that *E. coli* K12 adaptation to lettuce seedlings was accompanied by a dramatic increase in tolerance to oxidative stress caused by hydrogen peroxide. According to Queval et al. [20] leaf H_2_O_2_ concentrations in unstressed plants might range from 0.05 to 5.0 µmol×g FW^−1^. Thus, the concentration of H_2_O_2_ used in our study might be in the range that could be found in plant tissues and/or it might produce a level of oxidative stress on the bacterial cells comparable to that exerted by the colonized seedlings.

A microarray-based whole-genome transcriptional profiling and quantitative reverse transcription PCR of *E. coli* strain O157 exposed to lettuce lysates identified the up-regulation of genes for oxidative stress tolerance, among others. However, bacteria bearing a deleted copy of the *oxyR* gene (coordinating the response to oxidative stress) did not show reduced survival or growth over 5 h in lettuce lysates compared to the parental strain [21]. In agreement, the sole acclimation to oxidative stress appears not to be enough for *E. coli* to enhance its lettuce colonization efficiency, suggesting that adapted bacteria might display a more complex array of adaptations for enhanced fitness in/on the alternative host.

Plant-adapted *E. coli* also showed a stronger chemotactic response than non-adapted bacterial. Additionally, non-chemotactic bacteria were less competent for lettuce colonization. It had been shown before that root exudates are an important source of nutrients for the microorganisms present in the rhizosphere and participate in the colonization process through chemotaxis of soil microorganisms. It was further demonstrated that rice root exudates induce a higher chemotactic response for endophytic bacteria than for other bacterial strains present in the rice rhizosphere [22]. Mutational analysis conducted with the rhizospheric bacterium *Pseudomonas fluorescens* further demonstrated the requirement of the CheA-dependent chemotactic response for full colonization capacity of tomato roots. Although as competent for roots colonization as the wild type strain when inoculated alone in a gnotobiotic system, the *cheA*-defficient strains were 10- to 1000-fold less competent to colonize tomato roots in either the gnotobiotic system or in non-sterile potting substrate in competition assays together with the wild type strain [23]. Thus, it appears that human commensal *E. coli* are not only genetically equipped but also remains flexible enough to activate adaptive responses to signals from alternative hosts such as plants in an apparently similar fashion as bacteria displaying a preferred rhizospheric or endophytic lifestyle.

Hence, the findings of this study help to understand the physiology of *E. coli* during the adaptation process of colonization of alternative hosts such as lettuce seedlings.

## Materials and Methods

### Bacterial Strains and Culture Conditions

*Escherichia coli* K12 strain MG1655 was the reference strain used in this work. Strain MG1655 was transformed using pKEN2-GFPmut2 [24] or pDsRed2 plasmid (Clontech) encoding Green Fluorescent Protein (GFP) or Red Fluorescent Protein (RFP) respectively. For chemotaxis assays, the strains of *E. coli* K12 RP437 [25] and RP9535 (Δ*cheA1643*) [24] were used. *Escherichia coli* strains were cultivated overnight at 37°C in Luria Bertani broth (LB) or complete M9 medium with shaking at 150 rpm. Fluorescence-labeled strains were cultured in the presence of 100 µg×mL^−1^ ampicillin. For the standardized colonization assay (see below), inocula were prepared from bacterial cells cultivated in LB or M9 media and cells at the exponential phase of growth were collected by centrifugation at 6,000 rpm for 5 min at room temperature and rinsed with sterile 0.85% NaCl.

### Cultivation and Bacterial Colonization of Lettuce Seedlings

Lettuce (*Lactuca sativa* cv. criolla) seeds were provided by Pro-Huerta Program of Instituto Nacional de Tecnología Agropecuaria (INTA, Argentina). Seeds were disinfected with 95% (v/v) ethanol for 1 min and 0.36% (w/v) active chlorine for 3 min and then rinsed with sterile distilled water three times. Disinfected seeds were allowed to germinate onto 0.6% agarized Hoagland solution in Petri dishes and maintained for 48 h at 22±2°C under a photoperiod of 16 h of white light at 176 µmol photons×m^−2^×s^−1^. The effectiveness of disinfection was tested in parallel assays by incubating a sample of the pool of disinfected seeds into LB broth overnight at 37°C with shaking. Only seeds producing no microorganisms growth from these tests were kept for colonization assays.

For the standard colonization assay bacterial cells were inoculated into 20 mL of melted agarized-Hoagland-medium at 0.5X strength containing 0.6% agar at about 40°C to obtain a final titer of 10^8^ colony forming units (CFUs) ×mL^−1^ in 50 mL sterile test tubes. Inoculum size was first estimated by counting bacterial cells under a microscope using a Neubauer chamber. Samples of the inocula were used for confirmatory determinations of CFUs onto solidified LB medium. Since either GFP- or RFP-expression in the fluorescent-labeled strains is directed by the *E. coli lacZ* promoter, 10 µM IPTG was incorporated into the agarized medium for consistent strong fluorescence. After allowing the inoculated medium to solidify at room temperature, three equally developed two-day-old seedlings were aseptically transferred onto the inoculated medium of each test tube inside a transfer hood aided by sterile tweezers. It was noticed in preliminary assays that pooling three seedlings together produced a similar range of variation of *E. coli* loading of colonized seedlings than scoring individual seedlings. Thus, each data point of the standard assay consisted of three seedlings in order to simplify the preparation of multiple samples. For surface inoculation assays, seedlings were transferred onto non-inoculated agarized-Hoagland medium and when the first true leaf appeared, a droplet containing bacteria at 10^8 ^CFU×mL^−1^ was inoculated onto this leaf. The inoculated plants were placed in a growing chamber at 22±2°C under a photoperiod of 16 h of white light at 176 µmol photons×m^−2^×s^−1^ and analyzed 20 days after inoculation.

For each data point of CFUs from colonized seedlings, roots or leaves samples corresponding to the three seedlings from each test tube were pooled together, weighed and immediately homogenized in sterile 0.85% NaCl in a Potter-type tissue grinder. Samples were immediately spotted onto LB plates in triplicate for CFUs determination of each data point.

For fluorescence microscopy, samples were mounted directly onto glass slides and examined through a Nikon eclipse 600 fluorescence microscope. Images were captured with an Olympus DP72 camera. Samples were further examined using a Nikon confocal laser scanning microscope (Nikon C1 Plus with an Eclipse Ti Inverted Microscope).

### Competitive Index Assays

GFP-labeled non-adapted bacteria (cultivated in LB medium) were allowed to compete against RFP-labeled cells isolated from leaves. For confirmatory purposes reciprocal assays were run in which non-adapted cells were RFP-labeled and plant acclimated cells were GFP-labeled. Cells were co-inoculated into test tubes at a titer of 0.5×10^8 ^CFU×mL^−1^ of each strain (1∶1 ratio) to achieve the final titer of the standard colonization assay. Initially, bacterial doses were estimated by counting the cells of the suspensions under a microscope before inoculation of the test tubes with the mixed populations. Finally, samples of the suspensions were used to determine CFUs onto LB plates containing 10 µM IPTG, and the initial input ratio of GFP-labeled to RFP-labeled viable cells was determined. Adapted bacteria were obtained from ground colonized lettuce leaves (see standard colonization assay) and directly inoculated into the substrate of two-days-old seedlings without passages through laboratory media. After 20 days, post inoculation macerates were prepared from leaves to determine the output ratio of GFP-labeled to RFP-labeled viable cells. The competitive index (CI) was defined as the ratio between the output and the input ratios. A deviation from CI = 1 indicated that one of the strains outcompeted the other under the conditions imposed.

### Oxidative Stress Tolerance

Bacterial *Escherichia coli* cells cultured in LB medium or isolated from leaves (first cycle of colonization) were inoculated at 10^5^ to 10^6^ CFUs×mL^−1^ into M9 complete medium supplemented with 0; 0.5; 2; 8 or 32 mM H_2_O_2_. Cultures were incubated at 37°C with shaking at 150 rpm and CFUs were determined over time onto LB plates. As a control for *E. coli* acclimation to oxidative stress, cells were incubated in the presence of 8 mM H_2_O_2_ in M9 medium for 30 min and then survival in the presence of 10 mM H_2_O_2_ was scored over time.

To determine the effect of acclimation to oxidative stress on the ability of *E. coli* to colonize lettuce seedlings bacteria, inocula were prepared by treating cells cultured in LB medium with 8 mM H_2_O_2_ (sub lethal dose) or lettuce extract for 30 min before inoculation into lettuce seedlings.

### Chemotaxis Assays

The chemotaxis response of plant-adapted cells (isolated from colonized leaves) was evaluated by puncture inoculation of the cells onto TB solid medium (1 g peptone, 1 g NaCl and 0.25 g agar-agar per 1L of distilled water). Chemotaxis towards aqueous leaf extracts was assayed by the method proposed by Adler [27] with modifications [22]. To prepare leaf extracts, lettuce plants were purchased from a local store and young leaves were thoroughly rinsed with sterile distilled water. Aided by a scalpel, samples of the main vascular bundle or the remainder of the leaf (leaf blade) were taken and immediately ground in liquid nitrogen. To obtain the clarified extracts the thawed samples were subjected to 4 cycles of vortexing and incubating on ice for 5 minutes, and then centrifuged at 18,000 rpm at 4°C for 15 minutes. Finally, samples were sterilized by filtration through 0.22 µm membranes. The protein concentration in a typical preparation were 3.12 mg×mL^−1^ or 2.24 mg×mL^−1^ for leaf blade or main vein preparations, respectively. Sterile capillary tubes of 3 cm long with an internal diameter of 1 mm were plunged open end down into a 1.5 mL centrifuge-tubes containing 500 µl of lettuce extracts or phosphate buffered saline (PBS; 1.1 g K_2_HPO_4_, 0.32 g KH_2_PO_4_ and 8.5 g NaCl in 1L of distilled water, pH 7.2). Once the liquid ascended about 1.5 cm, the capillary was inserted into the 1.5 mL centrifuge-tubes containing the suspension of 10^7^ bacteria×mL^−1^. After 40 minutes of incubation, the capillary was removed and its exterior was rinsed with sterile 0.85% NaCl. The sealed end was broken off over a 1.5 mL tube containing sterile 0.85% NaCl. Serial dilutions were prepared and the CFUs were determined by counting onto LB agar plates. The plates were incubated at 36±1°C for 24 h. Finally, responses were calculated as the number of CFU per capillary containing lettuce extract, divided by the number of CFU in capillaries containing the control (PBS). *Escherichia coli* strains RP437 (wild type for chemotaxis analysis) and RP9535 (Δ*cheA*1643) were used as positive and negative controls, respectively.

### Statistical Analysis

Data are expressed as means or medians ± SD or as individual data points and the corresponding mean value. Colonization data were transformed to log_10_ prior to performing statistical analysis. The data shown in Fig. 4 were subjected to the Mann-Whitney test and those shown in Figs. 6 and 7 to a Wilcoxon test using the GraphPad statistical package.

## Supporting Information

Figure S1 **Laser confocal images of GFP-labeled** ***E. coli*** **K12 colonization of lettuce leaves.** Representative photomicrographs in the (A) XY and corresponding (B) YZ, or (C) XZ planes. In (B) and (C) data integration spans the complete width of the leaf. White bars correspond to (A) 20 µm or (B and C) 50 µm. Note that a single GFP-labeled *E. coli* aggregate spans the complete width of the leaf.
(DOCX)

Figure S2 **Representative fluorescence photomicrographs of** ***E. coli*** **K12-colonized lettuce leaves.** (A) Detail of single *E. coli* cells labeled with RFP in lettuce leaves at an early stage of colonization photographed at a magnification of 750X, and the white bar represents 10 µm. (B) Detail of bacterial aggregates in the proximity of vascular tissue in lettuce leaves at advanced stages of colonization. The picture was taken at a magnification of 150X and the white bar represents 20 µm.
(DOCX)

Figure S3 **Effect of acclimation of** ***E. coli*** **K12 to H_2_O_2_ on lettuce colonization.** (A) Survival of *E. coli* K12 with (▪) or without (▴) previous incubation in the presence of 8 mM H_2_O_2_ for 30 min and then transferred to 10 mM H_2_O_2._ Data represent the mean and SD of 2 independent assays. (B) Effect of acclimation of *E. coli* K12 to 8 mM H_2_O_2_ or leaves lysates for 30 minutes on its performance during colonization of lettuce leaves. No addition or freshly isolated cells from leaves were used as controls. Data represent the mean and SD of 3 independent assays.
(DOCX)

Figure S4 **Effect of initial** ***E. coli*** **K12 inoculum size and time course of colonization of lettuce leaves.** (A) Assays were run according to the standardized conditions at 20 days after inoculation (see materials and methods) except that the initial dose varied from 10 to 10^8^ cells×mL^−1^ of agarized medium. Data represent the mean and SD of 3 independent assays comprising a pool of all leaves from three seedlings from each assay. (B) Assays were run according to the standardized conditions (see materials and methods) except that data were separately collected for leaves (empty bars) or roots (full bars) at different times after inoculation. Data represent the mean and SD of 3 independent assays.
(DOCX)

Video S1 **Laser confocal video of GFP-labeled** ***E. coli*** **K12 colonization of a lettuce leaf.** The video integrates confocal images at 1 µm intervals starting from the adaxial face of the leaf. Note the stomata (just a few) plane at 0.19 min at the adaxial face; two GFP-labeled *E. coli* microcolonies: one at the left (0.23–0.33 min) and one to the right (0.29–0.42 min); a leaf vein longitudinal-section confocal plane at 0.31 min; and abaxial face stomata (more abundant) plane at 0.39 min. Both microcolonies are close to the vascular tissue of the leaf.
(MP4)
