# Supplementary material for: Plant-Adapted Escherichia coli Show Increased Lettuce Colonizing Ability, Resistance to Oxidative Stress and Chemotactic Response
Source: PLoS One. 2014 Oct 14;9(10):e110416. doi: 10.1371/journal.pone.0110416 (PMC4196987; doi:10.1371/journal.pone.0110416)
Supplement: Figure S1 — Laser confocal images of GFP-labeled E. coli K12 colonization of lettuce leaves. Representative photomicrographs in the (A) XY and corresponding (B) YZ, or (C) XZ planes. In (B) and (C) data integration spans the complete width of the leaf. White bars correspond to (A) 20 µm or (B and C) 50 µm. Note that a single GFP-labeled E. coli aggregate spans the complete width of the leaf. (DOCX) [file pone.0110416.s001.docx]

**Supporting Information**

**Plant-Adapted *Escherichia coli* Shows Increased Lettuce Colonizing Ability, Resistance to Oxidative Stress and Chemotactic Response**

**Dublan *et al***


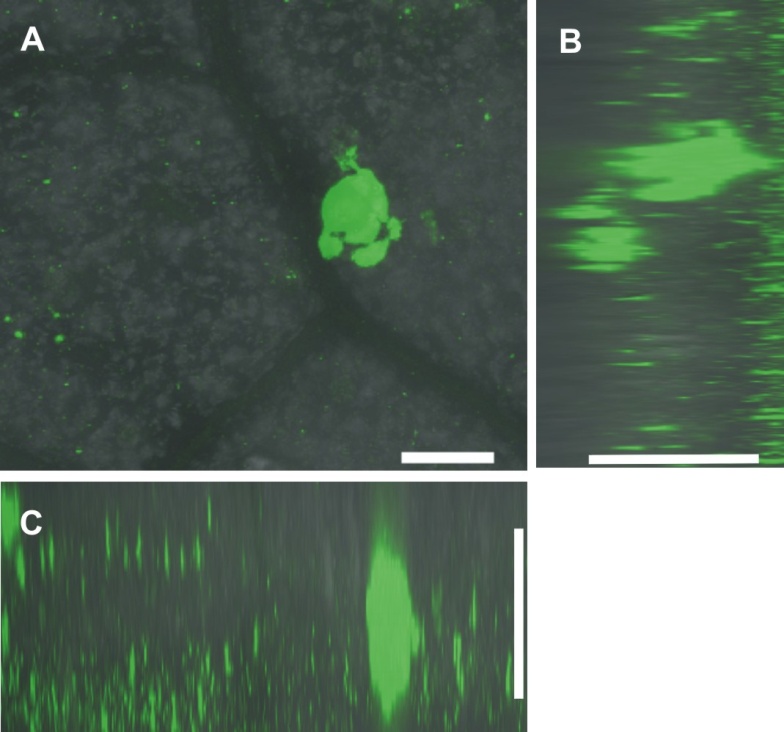


**Figure S1. Laser confocal images of GFP-labeled *E. coli* K12 colonization of lettuce**

**leaves**. Representative photomicrographs in the (A) XY and corresponding (B) YZ, or

(C) XZ planes. In (B) and (C) data integration spans the complete width of the leaf. White bars correspond to (A) 20 μm or (B and C) 50 μm. Note that a single GFP-labeled *E. coli* aggregate spans the complete width of the leaf.
