# Supplementary material for: Plant-Adapted Escherichia coli Show Increased Lettuce Colonizing Ability, Resistance to Oxidative Stress and Chemotactic Response
Source: PLoS One. 2014 Oct 14;9(10):e110416. doi: 10.1371/journal.pone.0110416 (PMC4196987; doi:10.1371/journal.pone.0110416)
Supplement: Figure S2 — Representative fluorescence photomicrographs of E. coli K12-colonized lettuce leaves. (A) Detail of single E. coli cells labeled with RFP in lettuce leaves at an early stage of colonization photographed at a magnification of 750X, and the white bar represents 10 µm. (B) Detail of bacterial aggregates in the proximity of vascular tissue in lettuce leaves at advanced stages of colonization. The picture was taken at a magnification of 150X and the white bar represents 20 µm. (DOCX) [file pone.0110416.s002.docx]

**Supporting Information**

**Plant-Adapted *Escherichia coli* Shows Increased Lettuce Colonizing Ability, Resistance to Oxidative Stress and Chemotactic Response**

**Dublan *et al***


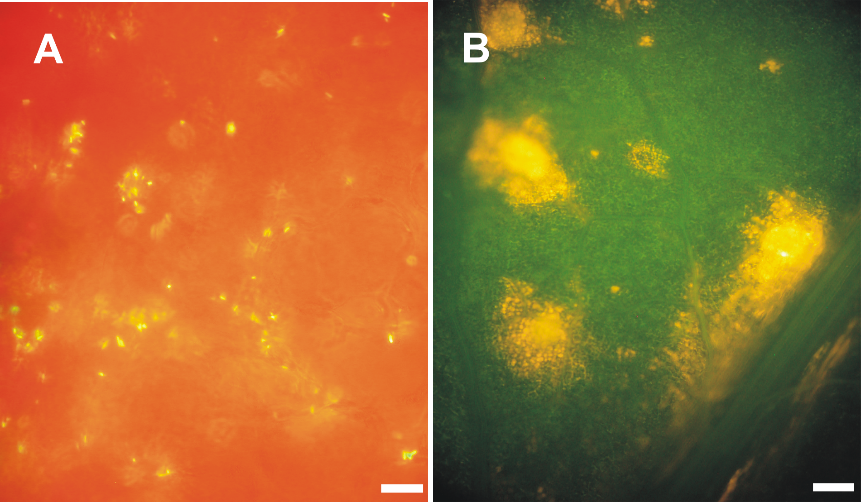


**Figure S2. Representative fluorescence photomicrographs of *E. coli* K12-colonized lettuce leaves**. (A) Detail of single *E. coli* cells labeled with RFP in lettuce leaves at an early stage of colonization (10 days after inoculation) photographed at a magnification of 750X, and the white bar represents 10 μm. (B) Detail of bacterial aggregates in the proximity of vascular tissue in lettuce leaves at advanced stages of colonization (20 days after inoculation). The picture was taken at a magnification of 150X and the white bar represents 20 μm.
