# Supplementary material for: Plant-Adapted Escherichia coli Show Increased Lettuce Colonizing Ability, Resistance to Oxidative Stress and Chemotactic Response
Source: PLoS One. 2014 Oct 14;9(10):e110416. doi: 10.1371/journal.pone.0110416 (PMC4196987; doi:10.1371/journal.pone.0110416)
Supplement: Figure S3 — Effect of acclimation of E. coli K12 to H2O2 on lettuce colonization. (A) Survival of E. coli K12 with (▪) or without (▴) previous incubation in the presence of 8 mM H2O2 for 30 min and then transferred to 10 mM H2O2. Data represent the mean and SD of 2 independent assays. (B) Effect of acclimation of E. coli K12 to 8 mM H2O2 or leaves lysates for 30 minutes on its performance during colonization of lettuce leaves. No addition or freshly isolated cells from leaves were used as controls. Data represent the mean and SD of 3 independent assays. (DOCX) [file pone.0110416.s003.docx]

**Supporting Information**

**Plant-Adapted *Escherichia coli* Shows Increased Lettuce Colonizing Ability, Resistance to Oxidative Stress and Chemotactic Response**

**Dublan *et al***


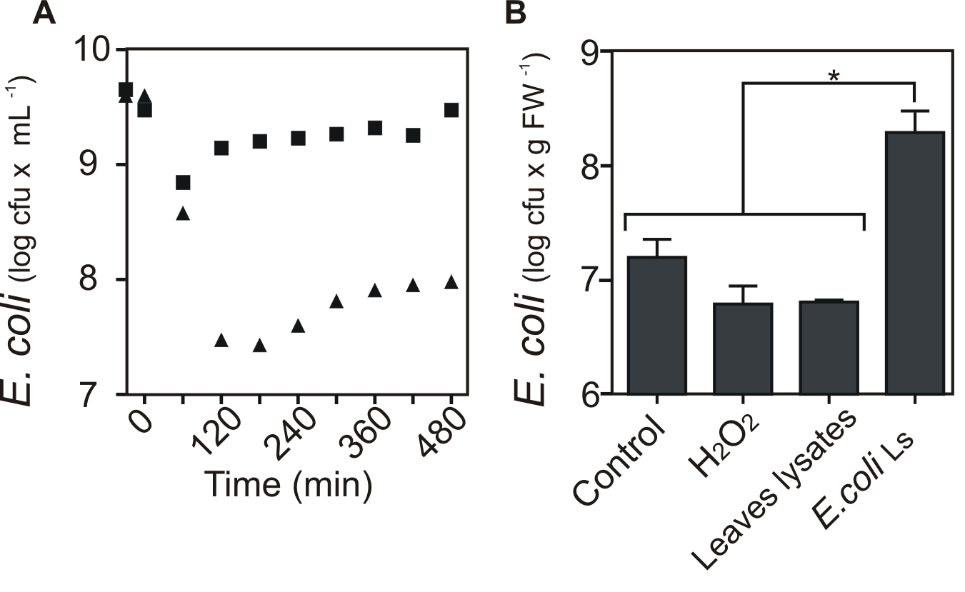


**Figure S3. Effect of acclimation of *E. coli* K12 to H_2_O_2_ on lettuce colonization.** (A) Survival of *E. coli* K12 with (■) or without (▲) previous incubation in the presence of 8 mM H_2_O_2_ for 30 min and then transferred to 10 mM H_2_O_2._ Data represent the mean and SD of 2 independent assays. (B) Effect of acclimation of *E. coli* K12 to 8 mM H_2_O_2_ or leaves lysates for 30 minutes on its performance during colonization of lettuce leaves. No addition or freshly isolated cells from leaves were used as controls. Data represent the mean and SD of 3 independent assays.
