# Supplementary material for: Plant-Adapted Escherichia coli Show Increased Lettuce Colonizing Ability, Resistance to Oxidative Stress and Chemotactic Response
Source: PLoS One. 2014 Oct 14;9(10):e110416. doi: 10.1371/journal.pone.0110416 (PMC4196987; doi:10.1371/journal.pone.0110416)
Supplement: Figure S4 — Effect of initial E. coli K12 inoculum size and time course of colonization of lettuce leaves. (A) Assays were run according to the standardized conditions at 20 days after inoculation (see materials and methods) except that the initial dose varied from 10 to 108 cells×mL−1 of agarized medium. Data represent the mean and SD of 3 independent assays comprising a pool of all leaves from three seedlings from each assay. (B) Assays were run according to the standardized conditions (see materials and methods) except that data were separately collected for leaves (empty bars) or roots (full bars) at different times after inoculation. Data represent the mean and SD of 3 independent assays. (DOCX) [file pone.0110416.s004.docx]

**Supporting Information**

**Plant-Adapted *Escherichia coli* Shows Increased Lettuce Colonizing Ability, Resistance to Oxidative Stress and Chemotactic Response**

**Dublan *et al***


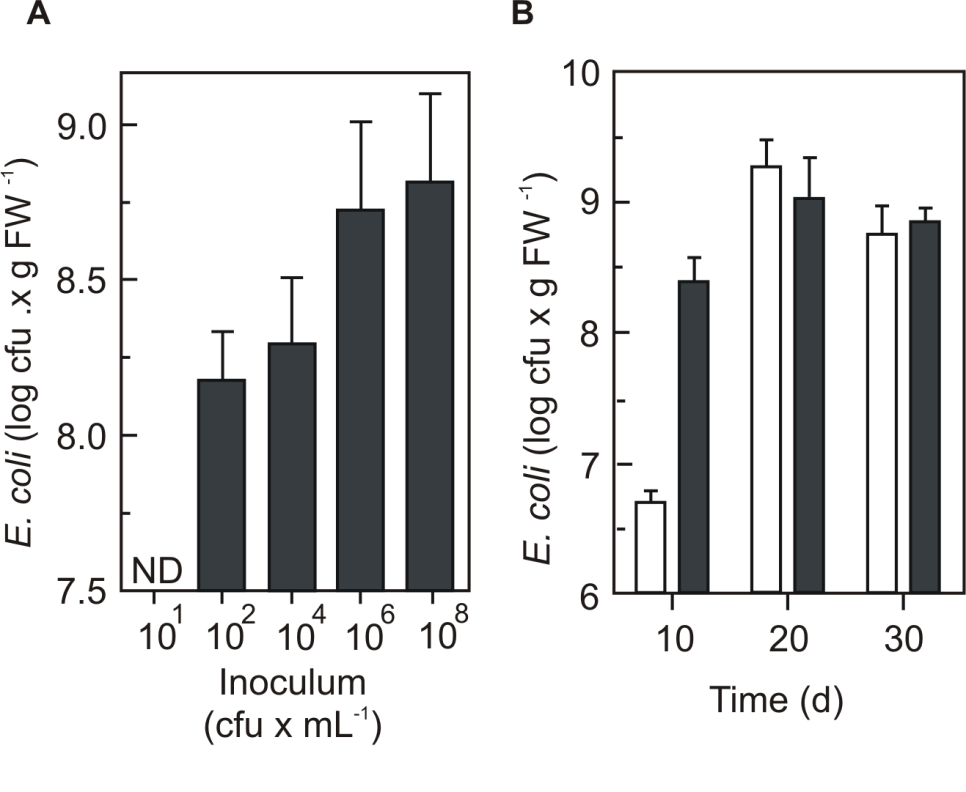


**Figure S4. Effect of initial *E. coli* K12 inoculum size and time course of colonization of lettuce leaves**. (A) Assays were run according to the standardized conditions at 20 days after inoculation (see materials and methods) except that the initial dose varied from 10 to 10^8^ cells x ml^-1^ of agarized medium. Data represent the mean and SD of 3 independent assays comprising a pool of all leaves from three seedlings from each assay. (B) Assays were run according to the standardized conditions (see materials and methods) except that data were separately collected for leaves (empty bars) or roots (full bars) at different times after inoculation. Data represent the mean and SD of 3 independent assays.
